# Characterization of difference in structure and function of fresh and mastitic bovine milk fat globules

**DOI:** 10.1371/journal.pone.0221830

**Published:** 2019-08-29

**Authors:** Aparna Verma, Tamoghna Ghosh, Bharat Bhushan, Gopinath Packirisamy, Naveen K. Navani, Pranita P. Sarangi, Kiran Ambatipudi

**Affiliations:** 1 Department of Biotechnology, Indian Institute of Technology, Roorkee, India; 2 Center for Nanotechnology, Indian Institute of Technology, Roorkee, India; Oregon State University, UNITED STATES

## Abstract

Characterization of milk fat globule (MFG) was performed to investigate the difference in MFG membrane (MFGM) between fresh and mastitis Holstein Friesian cow milk. Lipid distribution investigated by exogenous phospholipids using microscopy showed higher phospholipid content in fresh compared to mastitic MFGM. Xanthine oxidase assay indicative of membrane impairment revealed lower activity in mastitic samples compared to fresh globules. Of note, significantly higher roughness of globule surface and zeta potential was observed in mastitis compared to fresh globules. Influence of globule membrane on the interaction with *L*. *fermentum* demonstrated preferential adhesion of bacteria to fresh compared to mastitic globules including enhanced extent of binding. Results of the present study provides an insight of the interfacial changes occurring at the globule surface as well as highlighting the importance of selective bacterial interaction with milk components for the potential development of functional food with relevance to human health.

## Introduction

Bovine milk is a complex physiological fluid with multifaceted functionality made up of diverse set of proteins, lipids, carbohydrates and vitamins for the nourishment [1] and protection [2] of infants. Milk obtained from cows and buffaloes are useful in making a wide range of dairy products. In addition, bovine milk contains numerous health beneficial bioactives such as immunoglobulins, peptides, lactoferrin, growth factors, cytokines, nucleotides, oleic acid, conjugated linoleic acid and omega-3 fatty acids. Taken together, milk has evolved as a natural food and would be hugely beneficial to better understand these components to improve their positive effects on human health.

Milk fat remains suspended in aqueous phase as lipid droplets of triglycerides covered with a monolayer of phospholipids and an outer bilayer as spherical droplets called as milk fat globule (MFG) [3] and with its membrane known as MFG membrane (MFGM). The MFGM consists of a complex mixture of lipids (e.g. phospholipids), proteins (e.g. glycoproteins), cholesterol and enzymes [3], with nutritional, physiological and health benefits [4]. Due to these beneficial effects, there has been growing interest in improving MFG isolation and separation techniques, at laboratory and industrial scale, to minimize material losses of proteins and phospholipids for its incorporation into functional food [5]. Similarly, fat globule structure or membrane has also gained significant interest for probiotic delivery since lactic acid bacteria (LAB) have been observed to be located at the fat globule surface [6, 7], and over time get embedded in the MFGM, or enter the fat globules [8]. Due to the preferential adhesion and interaction of bacteria with MFGM combined with the resistance of fat globules to gastric acid and formation of physio-chemical barrier, it enables bacteria to reach the intestine in functional state and influence microbial colonization [9–11]. Although recent studies have emphasized the characterization of bacterial surface to influence the interaction of LAB with different MFG components [12, 13], little is known about the impact of alterations in globule surface components on the interaction with probiotic bacteria. In fact, some studies have reported the influence of temperature, pH, surface roughness, surface free energy of matrix/bacteria, hydrophilic and hydrophobic character of bacteria/matrix, surface charge, pilli on the bacterial surface [14, 15] and presence of surface glycoproteins like MUC 1 [16] on bacterial adhesion. Thus, it will be informative to study the influence of globules and its membrane in facilitating bacterial adhesion.

The aim of the present study was to perform a comparative study of fat globule surface between fresh and mastitic bovine milk. As a proof-of-concept, globules from mastitic milk was taken as a model system, reported to have loss in membrane components [17, 18] and/or unavailability of adhesive sites [19] and compared to globules from fresh milk of healthy Holstein Friesian (HF) cow. Consequently, analysis was performed to investigate (i) variation in the distribution of globule membrane lipids by exogenous phospholipids using confocal laser scanning microscopy (CLSM), (ii) loss in membrane-bound xanthine oxidase (XO) activity and, (iii) the interaction of *L*. *fermentum*, a common probiotic bacteria, with fresh and mastitic globules by microscopy and flow cytometry. Results of the present study show significantly lower membrane-bound XO activity in mastitic globules compared to fresh milk. Observations by fluorescent and scanning electron microscope (SEM) revealed preferential adhesion of *L*. *fermentum* to fresh MFGM including enhanced extent of binding by flow cytometry compared to mastitic globules highlighting the importance of selective interaction of probiotic bacteria with the globule membrane components.

## Materials and methods

### Whole milk collection

Milk samples from healthy (n = 10) and mastitic (n = 10) HF were collected in their first lactation stage (45–60 days) from dairy farms of Roorkee, India after taking informed consent from the farm owner. Healthy animals had somatic cell count (SCC) in the range of 1.1–1.5 x 10^5^ cells/ml, while animals confirmed positive for mastitis by California Mastitis Test (CMT) (DeLaval Pvt. Ltd, Pune, India) and bacteriological identification had SCC in the range of 5–10 x 10^6^ cells/ml determined by automated cell counter (DeLaval Cell Counter DCC, Tumba, Sweden) according to the protocol described by Schalm et al [20]. Furthermore, mastitic milk samples (n = 10) were subjected to bacteriological identification by isolating the infecting bacterial pathogen, *S*.*aureus* according to previously reported protocols [21]. The isolated bacteria were identified based on culture characteristics, staining and biochemical reactions at the genus level as reported by Quinn et al [22]. The farmer collected milk samples for routine diagnostic purposes with part of the samples used for experimentation and thus no approval for institute animal ethics committee was required for the study. All milk samples were collected into sterile tubes and processed fresh to minimize the problems associated with refrigeration causing no damage to the MFG surface.

### Isolation of milk fat globules

Milk samples collected from each animal was used as a biological replicate and analyzed in triplicates. To normalize the difference in globule number and individual variation, equal amount of cream (approximately 0.3 gm) containing the MFG was separated from each animal within a group (e.g. fresh and mastitis). In brief, 15 ml of whole milk was centrifuged at 3000 rpm (1157×g) at 25°C for 15 min for the separation of cream and skim milk. Following centrifugation, the cream was divided into three aliquots, with one aliquot resuspended in 1X PBS for observation of MFG under confocal microscope, second aliquot was used for topographical surface characterization of MFGM by atomic force microscopy (AFM) and zeta potential measurements, and the third aliquot was used to study interaction of *L*. *fermentum* with MFGM.

### Microstructural analysis of milk fat globules

The microstructural studies of the MFGM were evaluated using an inverted Axio Observer Z1 Zeiss LSM 780 by confocal laser scanning microscopy (CLSM) (Carl Zeiss, Jena, Germany). Experiments were performed using He-Ne633; He-Ne594 and He-Ne lasers operating at 543nm wavelength excitation (emission was detected between 565 and 615 nm).

The staining of phospholipids was performed as previously reported [23]. Briefly, the fluorescent dye N-(Lissamine rhodamine B sulfonyl) di-oleoyl-phosphatidylethanolamine (Rh-DOPE; Avanti polar lipids Inc., Birmingham, England), for labeling phospholipids on the membrane was prepared by dissolving the powder in chloroform at 10 μg/ml concentration. The cream samples resuspended in 1X PBS buffer were stained with Rh-DOPE in a ratio of 1:100 (v/v) and kept at room temperature for 20 min before performing microstructural analysis. Three independent biological experiments in triplicates were performed by taking 20 μl of the MFG stained with the fluorescent dye was deposited onto the glass, slowly mixed with low melting agarose (50 μl, 0.5% w/v in deionized water) (Sigma, Aldrich, St Louis, USA) and observed under 63x oil immersion objective.

Microscope with 63x/1.40 oil DIC M27 was used to visualize fat globules. The images with 512 X 512 pixels were converted into micrometer using Image J software version 1.64r (National Institutes of Health, Bethesda, USA). Globule size measurement was performed from 3 animals in each group (e.g. fresh and mastitis). Snapshots from five different fields with 25 globules in each field were selected to calculate the diameter of the MFG in each sample. The area of globules (4πr^2^) was calculated using Image J software tool in square microns.

### Fluorescence measurements and phospholipid quantitation of milk fat globules

For quantitative difference in phospholipids present on the globule membrane between both animal groups, fluorescence excitation spectra of cream samples were acquired in a range of 560–700 nm at 1 min interval using F-4600 Fluorescence Spectrophotometer (Hitachi, Tokyo, Japan) equipped with a Xenon lamp. The excitation and emission slit widths were both set to 5 nm with emission wavelength at 592 nm. In brief, cream was isolated from 10 ml of milk from fresh (n = 5), and mastitis (n = 5) HF cows and labeled by incubating the samples with Rh-DOPE (10 μg/ml) for 20 min. Subsequently, the samples were centrifuged and thoroughly washed to remove excess unbound dye and measurements were recorded after diluting each sample in 1X PBS buffer (pH 7). Three independent biological experiments in triplicates were performed and analyzed by spectrophotometer.

Total phospholipid in fresh and mastitic MFG were measured calorimetrically [24] by extracting lipid from equal amount of cream from all animals as described by [25]. The chloroform layer containing the phospholipids was collected and mixed with ammonium ferrothiocyanate solution resulting in the formation of red colour complex measured at 488nm. The final fluorescence of fresh and mastitic globules were calculated by subtracting the fluorescencent measured from membrane loss.

### Difference in xanthine oxidase activity between fresh and mastitic milk fat globules

To investigate the loss of globule membrane integrity, XO assay [26] was performed as reported earlier to determine the loss in activity of membrane-bound enzyme in fresh and mastitic MFG. In brief, cream containing the MFG was isolated by centrifuging whole milk (15 ml) at 3000 rpm at 25°C for 15 min and resuspended in 50 mM (1.9 ml) potassium phosphate buffer pH 7.5. The reaction mixture was made by addition of resuspended cream in 0.15 mM (1ml) xanthine solution and the formation of uric acid through the catalysis of xanthine by XO present in the MFGM was observed at 290 nm by recording the spectra after 30 min incubation at 25°C. As control, xanthine was replaced with buffer. These assays were conducted in biological triplicates and performed three times independently.

### Atomic force microscopy measurements and zeta potential of milk fat globule surface

The difference in topographical features of the MFG membrane between fresh and mastitic globules was confirmed by AFM with few modifications as reported earlier [27]. Images of the MFGM were collected in semi-contact imaging mode, where the probe taps on the surface at regular intervals with SPM NTEGRA system (NT-MDT, Russia). Ten microliters of freshly prepared MFG in 1X PBS buffer was used for imaging. The images were taken after the samples were heat fixed and completely air-dried. A silicon nitride coating cantilever of length 100 μm and force contact 5.5–22.5 N/m at a frequency of 1.01 Hz with pyramidal geometry was used to generate the images. The sample surface was examined in biological triplicates and repeated thrice to confirm the topology. All images were processed by using Nova software (1.026.1424 version).

Alteration of zeta potential indicative of change to the MFG membrane surface of fresh and mastitic MFG was measured as reported earlier [28] using a Zetasizernano ZS90 (Malvern Instruments Ltd, Malvern, U.K.) by determining the electrophoretic mobility based on electrophoretic light scattering. The electrophoretic mobility is converted to zeta potential using the Smoluchowski equation:

ζ=⋃η∕ε

where *ζ* is zeta potential, ⋃ is electrophoretic mobility, *η* is medium viscosity and *ε* is the dielectric constant. The samples for the measurement of zeta potential were prepared by resuspending the cream in 20 mM imidazole, 50 mM NaCl, 5 mM CaCl_2_ buffer at pH 7. Equal amount of cream was diluted to ten-fold in the buffer and measured in biological triplicates and average of three technical measurements were reported as zeta potential in millivolts (mV).

### Molecular characterization of *L*. *fermentum*

The probiotic bacterium *L*. *fermentum* NKN51 was isolated from yak cheese sampled from Indian Himalayan ranges. The strain NKN51 was characterized as a probiotic bacterium producing phytase enzyme [29]. Taxonomic confirmation of *L*. *fermentum* NKN51 by 16S rRNA sequencing was carried out with universal primers for amplifying 16S rRNA gene [5’- AGAGTTTGATCMTGGCTCAG -3’ (27F, forward primer) and 5’- CGGTTACCTTGTTACGACTT -3’ (1492R, reverse primer)]. The sequence obtained was aligned with reference to 16S-rRNA gene sequences of the GenBank using the Basic Local Alignment Search Tool (BLAST) algorithm available at National Centre for Biotechnology Information. The partial 16S rRNA gene sequence of *L*. *fermentum* NKN51 has been deposited in the GenBank database under the accession number KP244308.

Fluorescence microscope (Carl Zeiss, Axio Scope A1, Germany) using green (Excitation: 450–490 nm, Emission: 510 nm) and red (Excitation: 545/25 nm; Emission: 605/70 nm) filters at 1000x magnification (oil immersion objective W Plan-Apochromat 100 × /1.0 VIS-IR lens and 10x ocular lens, Carl Zeiss AG, Germany) was used to observe the interaction between MFG and bacteria. Images were captured and acquired using AxioCam MRc5 and acquisition software Axio Vision v.4.4 (Carl Zeiss, Gottingen, Germany).

### Investigation of bacterial adhesion to milk fat globules

For all experiments, *L*. *fermentum* cultures were inoculated 1:100 (v/v) in 10ml MRS broth (Himedia) and Mueller Hinton medium (Himedia), respectively and allowed to grow until the absorbance (OD) reached at 600 nm (A_600_) of 0.5, equivalent to 10^8^ CFU/mL before harvesting at log phase. The bacteria were washed twice with 1X PBS buffer by centrifuging at 2000 rpm for 5 min. To observe the interaction between *L*. *fermentum* and MFG, fresh and mastitic, two aliquots were prepared with one aliquot used to check for bacterial adhesion using fluorescence microscopy, while second was checked using SEM. Fluorescent and SEM experiments were performed in two separate experiments in triplicates.

#### Fluorescent microscope

Bacteria and fat globules (fresh and mastitis) were stained separately. Fat globule membrane was stained by Rh-DOPE (10 μg/ml in chloroform) for labeling phospholipids in a ratio of 1:100 (v/v), while bacteria were stained by acridine orange (AO) (10 mg/ml in water at a ratio of 1:1000) [13]. Subsequently, equal volumes of labeled bacteria and MFG were mixed and allowed to incubate for 15 min and were mixed with agarose (0.5% w/v) in 1:2 ratio and transferred to cover slipped slides for visualizing the interaction under microscope.

#### Scanning electron microscopy

Unstained *L*. *fermentum* bacterial cells and MFG samples from fresh and mastitis were mixed in equal proportion after 1:5 dilution with 1X PBS and incubated for 15 min at 37º C. After incubation, 10 μl of each sample was loaded on the glass slide and air-dried. Subsequently, samples were fixed with 2.5% glutaraldehyde for 1 h at room temperature followed by gentle gradual dehydration with ethanol (25%, 50%, 70%, 80%, 90%, and 100%). The prepared samples were dried and sputter coated with gold in BAL-TEC SCD 005 gold sputtering unit and observed by FE-SEM QUANTA 200 FEG, FEI Netherlands. The images were captured at 10,000X magnification in triplicates.

### Confirmation of bacterial adhesion to milk fat globules by flow cytometry

To investigate the extent of bacterial binding to fat globules, sucrose density gradient (SDG) separation was used to determine the adhesion efficacy of *L*. *fermentum* to MFGM) as reported earlier [13] followed by analyzing the interaction using fluorescence activated cell sorter (FACS). SDG consisted of equal volumes of lower 60% sucrose layer and upper layer of 20% sucrose in 2 ml micro-centrifuge tube. MFG samples of fresh and mastitis were pre-warmed at 37ºC for 10 min before mixing with *L*. *fermentum* labeled with acridine orange. Equal volumes of the bacteria and fat globules from each group were loaded on top of the gradient and incubated for 15 min at 37ºC, while stained bacteria were taken as control. The samples were centrifuged at 15,000 rpm for 10 min at room temperature. Subsequently, after centrifugation topmost layer of 20% sucrose was recovered and diluted in a ratio of 1:4 with 1X PBS. The diluted samples were analyzed by BD FACS Verse (San Jose, California, USA) flow cytometer and data were analyzed using BD Fuchsite software. Three independent biological experiments were carried for calculating mean fluorescent intensities of each group.

### Statistical analysis

Experimental values are reported as averages ± standard error of the mean (SEM) of biological and technical replicates. Data were analyzed by the unpaired two-tailed Student’s *t* test (Mann-Whitney and Wilcoxon). Statistical analysis was done using Graph Pad Prism, Version 6.01 GraphPad Software, Inc., La Jolla, USA. Results are reported with 95% confidence intervals.

## Results

### Isolation, characterization and microstructural analysis of bovine milk fat globules

**Fig 1:**
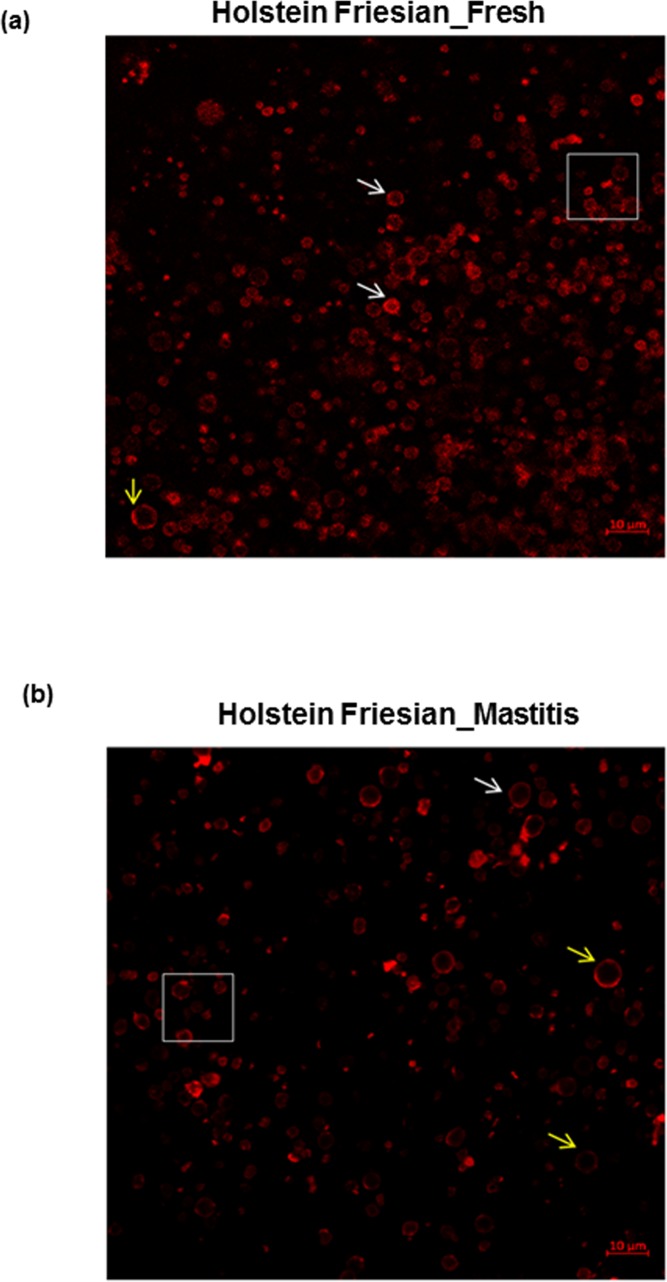
Milk fat globule shape and size characterization. Non-homogeneous distribution of polar lipids are shown by arrows, non-fluorescent regions rich in sphingomyelin appearing as black areas indicated as (yellow arrow) while globules with different fluorescent distribution on the periphery of fat globules indicated as (white arrows) using emission fluorescence of phosphatidylethanolamine-lissamine rhodamine by confocal laser scanning microscopy in Holstein Friesian cow (a) fresh (b) mastitis.

**Fig 2:**
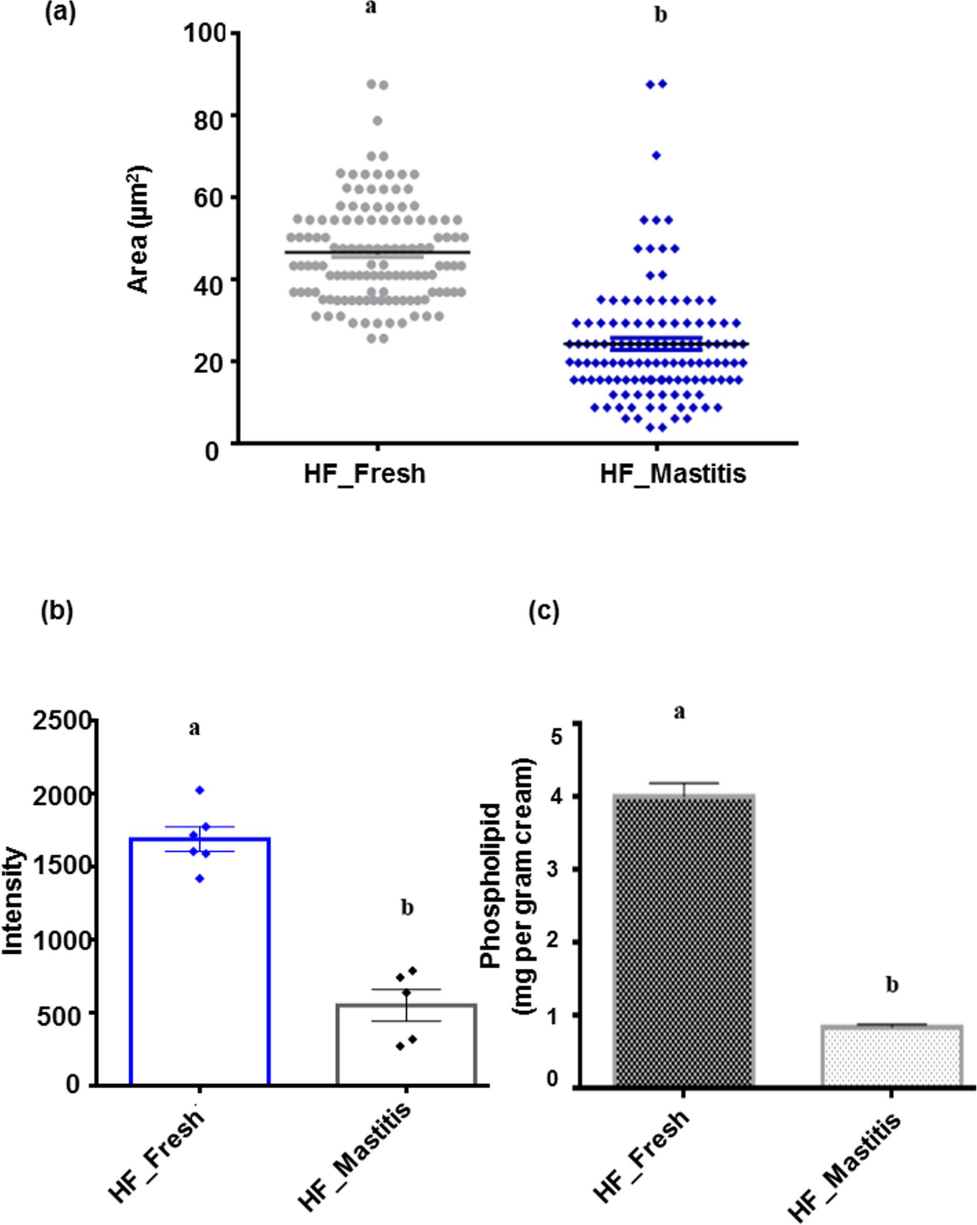
Heterogeneous distributions of fat globules and quantification of phospholipids in Holstein Friesian cow. (a) comparative analysis of the variation in size of fat globules is determined using differential interference contrast between fresh and mastitic globules. Dots represent globules from five different fields with 25 globules from each field in a sample (n = 125), and (b) comparative fluorescent measurements between fresh and mastitic samples, and (c) calorimetric phospholipid determination of polar lipids at 488 nm of fresh with reference to mastitic samples. Bars with different letters indicate significant difference (p ≤ 0.0001; averages ± SEM; Student’s t- test). Representative data of three independent biological experiments performed in triplicates. Scale bar = 10μm. Diameter of globules (n = 125) was used to calculate the area.

Emission fluorescence of fresh and mastitic globules stained with Rh-DOPE showed homogeneous distribution on the MFGM with non-fluorescent regions rich in sphingomyelin appearing as black areas indicated by arrows (yellow) (Fig 1A and 1B). Interestingly, two globules of the same diameter have difference in fluorescence on the globule surface as shown by arrows (white) (Fig 1A and 1B). The size of fat globules from both groups was calculated from the DIC images with significantly bigger globules (25.6 μm^2^ to 87.6 μm^2^) observed in fresh compared to mastitis (3.8 μm^2^ to 70.25 μm^2^) (Fig 2A), possibly due to change in fat synthesis during the secretion from epithelial cells [30].

Higher relative fluorescence intensities were observed in fresh globules (Fig 2B) corresponding to the presence of higher phospholipid content, while significantly lower (approximately 3 fold) intensity was observed in mastitic globules. Total phospholipids in both the groups (Fig 2C) calculated as per gram of cream showed significantly higher phospholipid content in fresh compared to mastitic samples (3.99±0.54 mg vs. 0.73±0.05 mg, respectively; p ≤ 0.0001).

### Membrane impairment indicated by lower Xanthine oxidase activity and higher surface roughening by atomic force microscopy

**Fig 3:**
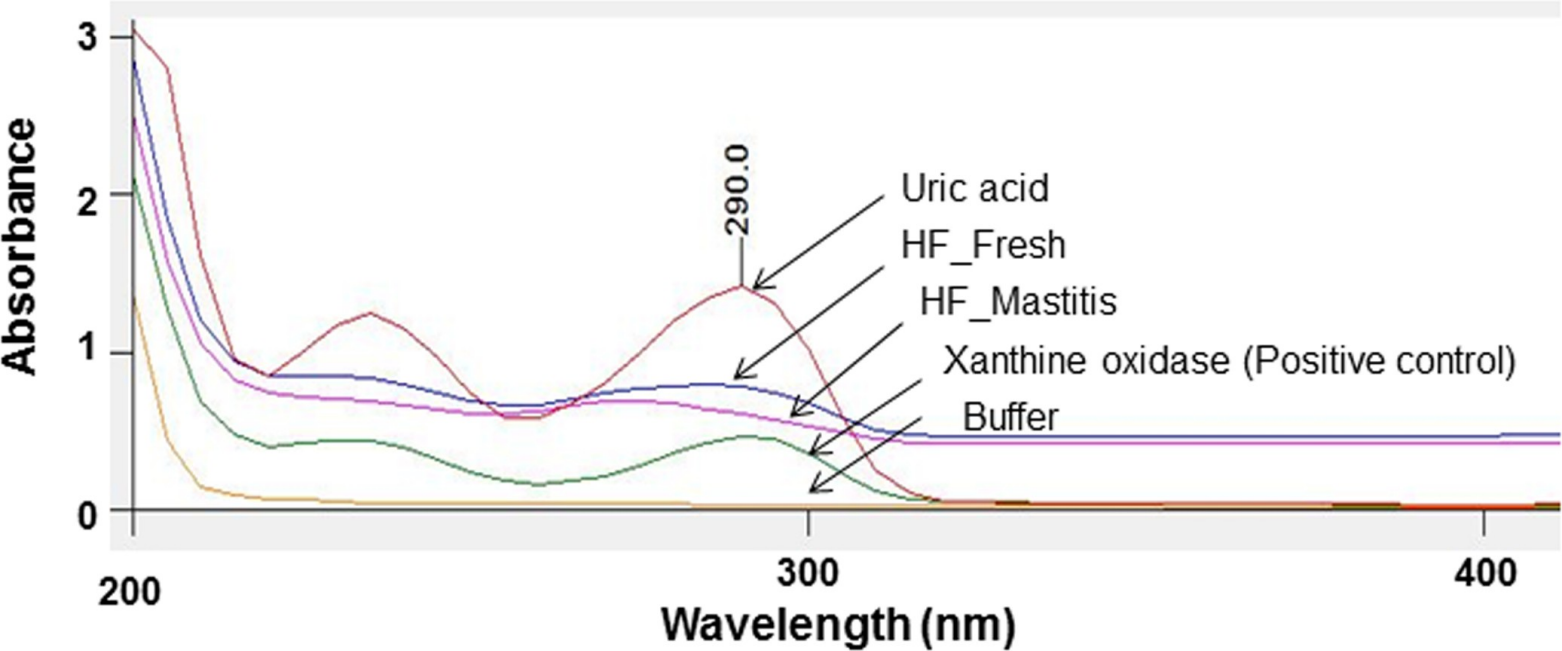
Detection of uric acid formation by UV absorption spectroscopy in fresh and mastitic Holstein Friesian globules. Spectral overlay of standard uric acid and uric acid formed by purified xanthine oxidase (positive control) in fresh and mastitic globules measured at 290 nm. Representative data of three independent biological experiments performed in triplicates.

**Fig 4:**
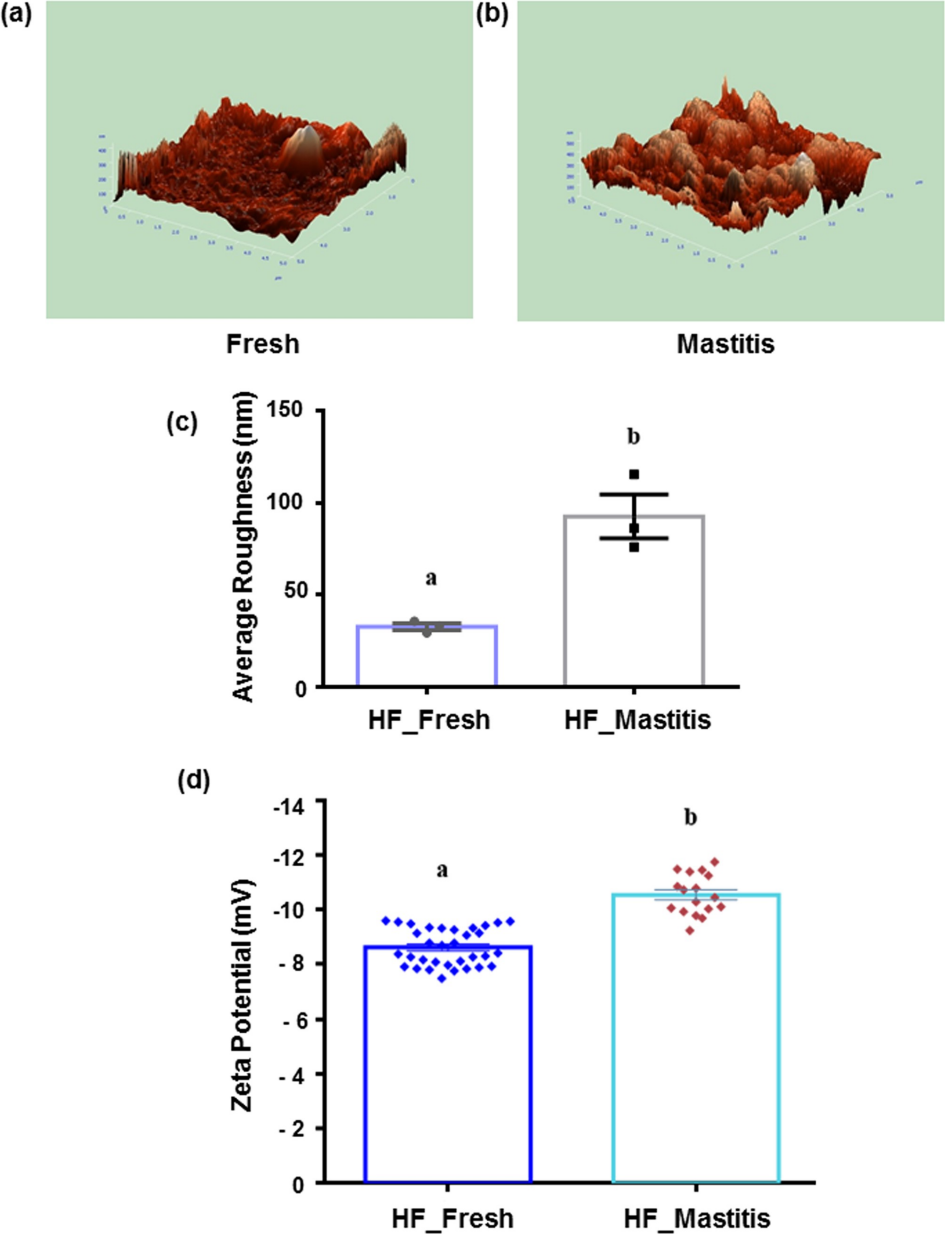
Surface characterization of milk fat globules between fresh and mastitic Holstein Friesian milk fat globules. Representative 3D projection of surface roughness by atomic force microscopy in (a) fresh (b) mastitis (c) surface roughness comparison between fresh and mastitic globules performed three times at different regions. Representative image of three independent biological experiments performed in triplicates, and (d) zeta potential measurements between fresh (-8.6±0.65 mV) and mastitic globules (-10.5±0.75 mV). Different letters indicate significant difference (p ≤ 0.001; averages ± SEM; Student’s t- test).

To establish a correlation between membrane damage resulting in reduced enzyme activity, XO assay was performed to measure the formation of uric acid due to the catalysis of xanthine by XO at 290 nm. Fresh globules with significantly higher absorbance indicating higher activity of bound enzyme compared to mastitis (Fig 3). Surface morphology of fresh globules (Fig 4A) was relatively smoother with lower roughness compared to mastitic globules (Fig 4B). The associated 3D projection (Fig 4A and 4B) reveals the surface characteristics of fresh MFG with root mean square of 42.60 nm, while the altered MFGM surface in mastitis was detected with root mean square of 148.41 nm. The topology measurements were confirmed by repeating three times in different regions with significant (p ≤ 0.04) difference observed between fresh (32.7) and mastitic (92.54 nm) globules (Fig 4C). The zeta potential measured as a possible indicator of the degree of change to the fat globule’s surface (Fig 4D) demonstrated significant (p ≤ 0.0001) difference between fresh (-8.6±0.65 mV) and mastitis (-10.5±0.75 mV), indicating difference in globule surface charge between the two conditions.

### Selective binding of *L*. *fermentum* to milk fat globule membrane

**Fig 5:**
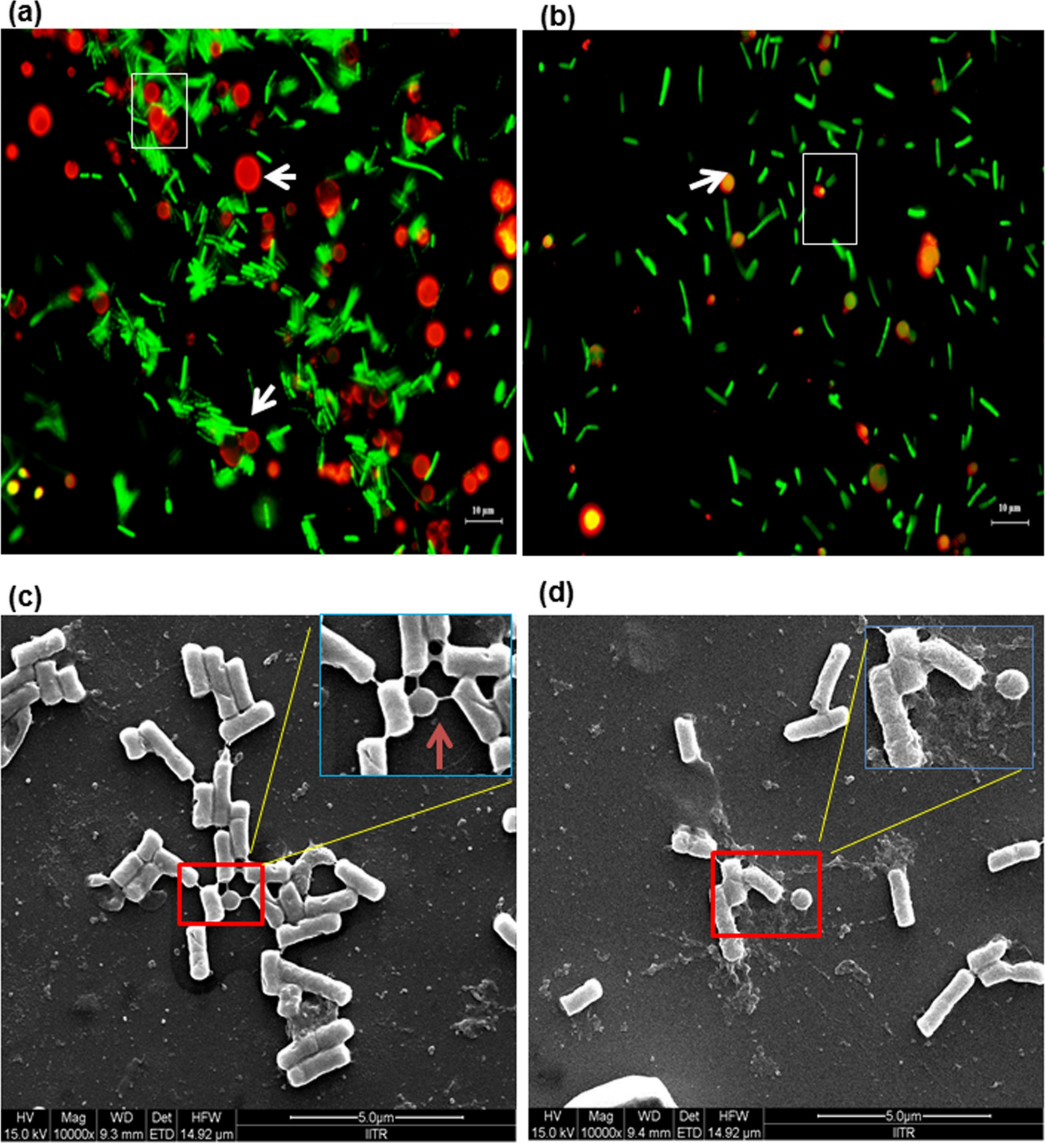
Interaction of *L*. *fermentum* with milk fat globules of fresh and mastitic globules of Holstein Friesian cow by microscopy. Preferential and selective interaction of *L*. *fermentum* NKN 51 with milk fat globules (MFG) (a) fresh (b) mastitis. Adhesion of bacteria to milk fat globule is delineated with square, while fresh and mastitic globules are shown by white and yellow arrows, respectively. The globules were stained with phosphatidylethanolamine-lissamine rhodamine (red), while bacteria were stained with acridine orange (in green). Selective interaction was observed in all two independent experiments in triplicates. Scale bar = 10 μm. SEM micrograph of interaction of unstained *L*. *fermentum* with (c) fresh, and (d) mastitic fat globules at (10,000X). Delineation shows the interaction between bacteria and globule. Representative images of three independent biological experiments performed in triplicate.

Compared binding preference of *L*. *fermentum* to fresh and mastitic globules showed preferential adhesion of *L*. *fermentum* towards fresh MFGM was observed with greater numbers of bacteria observed around the globule membrane stained in acridine orange shown by box (white) (Fig 5A). In contrast, bacterial adhesion was not uniform and less pronounced to mastitic globules (Fig 5B). Smoother globular membrane shape in fresh MFG is indicated by arrow (white, Fig 5A), while relatively rough membrane of fat globules in mastitis was detected as shown by arrow (yellow, Fig 5B). Thus, preferential adhesion of bacteria highlights the importance of fresh fat globule membrane proteins and phospholipids in promoting selective binding of *L*. *fermentum* to fresh MFGM.

The adhesion of *L*. *fermentum* with globules using SEM demonstrated interaction (indicated by red box) (Fig 5C), while green arrows show the adhesive linkage between the globule and bacteria. In contrast, no adhesive interaction to the surrounding bacteria was observed with mastitic globules (Fig 5D).

**Fig 6:**
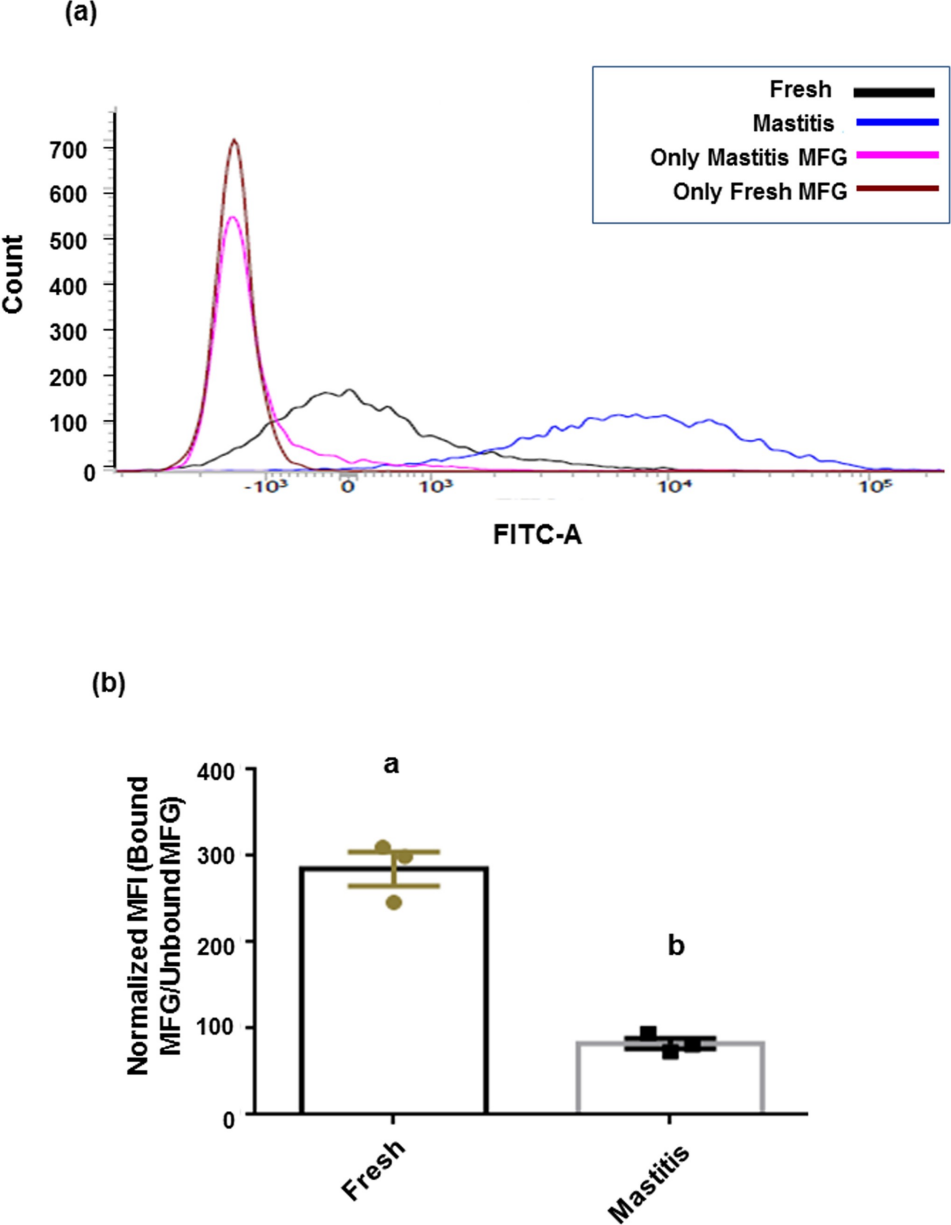
Interactive behavior of *L*. *fermentum* with fresh and mastitic globules of Holstein Friesian cow by flow cytometry. (a) Histogram overlay of bacteria bound to fresh and mastitic globules, Unbound MFGs of each group are taken as negative controls and only bacteria are taken as positive control. (b) The bar diagrams represent the mean fluorescent intensity of each group with significantly (p ≤ 0.001) higher mean fluorescent intensity in fresh globules than mastitic. The bar graph represents a ratio of mean fluorescent intensity of bound and unbound MFGs in each group. The data represents the averages of three independent biological experiments.

Extent of binding of bacteria to fat globules was further confirmed using flow cytometry. The histogram overlay (Fig 6A), represents the binding of acridine orange stained *L*. *fermentum* with fresh and mastitic globules along with their respective unbound MFGs. Fresh globules showed three fold higher mean fluorescent intensity (MFI) compared to mastitic globules. Furthermore, the shift in the fluorescent intensity was significantly (p ≤ 0.001) higher in fresh globules as represented in the bar diagram (Fig 6B). The bar graph represents a ratio of MFI of bound and unbound MFGs in each group.

## Discussion

Natural emulsions such as milk and its components have been an important source to deliver nutrients to promote human health. More recently, there has been a growing interest in the interaction of specific milk components such as MFGM and LAB due to their ability to form physiochemical barrier and resist digestion, enabling to passage of probiotic bacteria to the intestine intact [10, 11] to promote immune system and microbial colonization [31]. Similarly, MFGM has also been used in food to inhibit bacterial and viral binding to intestinal mucosa [32]. However, limited information is known about the way LAB interacts with MFGM and the impact of globule integrity has on bacterial interaction.

Previous studies have reported isolation and characterization of the MFGs by DIC and CLSM in different species such as buffalo [33] and cow [31, 34]. However, this study, to the best of our knowledge, is the first to report the differences in globule membrane between fresh and mastitic milk and the impact of MFGM surface morphology has on bacterial interaction. Fat globules in both groups showed variation in globule size with smaller globules observed in mastitis compared to fresh, possibly due to the disease affecting the milk producing machinery [17]. Of note, non-homogenous distribution of fluorescence was observed in both groups indicative of heterogeneous distribution of phospholipids around the periphery of the globules. Nevertheless, such heterogeneities were previously reported in human [35] and bovine MFG [36], potentially due to the presence of sphingomyelin rich domains in the bilayer of MFG [37]. Significantly lower emission fluorescence and low phospholipid content was detected in mastitis than fresh samples, suggesting an alteration in the membrane surface component [38].

Biological study performed to determine loss of membrane components using membrane-bound XO assay demonstrated lower enzyme activity in mastitic globules, indicative of altered globule membrane architecture resulting in dissociation of the enzyme from the membrane, compared to fresh globules. Lower enzymatic activity in mastitic MFG was consistent with a previous study that reported association of membrane impairment to lower activity due to dissociation of the enzyme from the membrane [17]. Xanthine oxidase is one of the most abundant enzymes [39] bound to the membrane and any damage to the integrity of the MFGM would results in decrease in enzymatic activity that can be measured by a spectrophotometer assay with minimal variation. Subsequently, difference in nanoscale architecture of MFG’s surface was confirmed by AFM topography, which indicated relatively smoother surface morphology in fresh globules compared to mastitis, possibly due to higher proteolysis and lipolysis of milk [18]. In parallel, significant decrease in zeta potential measurements was observed in mastitic relative to fresh globules, indicating possible loss of membrane component in line with previous reports [28]. Nevertheless, lowering of zeta potential could also be due to lower levels of phospholipids including loss (10%) [17] in the MFG membrane in impaired as well as change in the surface charge due to binding of plasma milk proteins on to the surface [28].

Probiotic bacteria such as *Lactobacillus* spp. are used to maintain a balance of beneficial bacteria in the intestine and improve our immune system [40]. These probiotic bacteria preferentially interact with different dairy components such as MFG and its membrane acting as delivery vehicles [13]. In fact, some studies have reported the influence of different milk components on specific gene expression of LAB associated with binding [41]. Interaction of LAB with different milk components provides protection during storage and transfer through stomach (e.g. pH change) [13]. For example, MFGM has been recently used as a transfer vehicle due to its ability to resist gastric juices and shield the probiotic bacteria *en route* to intestine in viable state for maintaining physiological gut micro flora [10]. However, limited studies have investigated the impact of MFGM surface would exert on its interaction with LAB to better understand the influence of MFG’s membrane component on probiotic bacteria [42]. Consequently, binding studies were performed to compare the interaction of *L*. *fermentum* with MFGM isolated from fresh and mastitic samples. Interestingly, preferential binding of LAB was observed with fresh MFGM compared to mastitic globules, suggesting distinct binding specificity of *L*. *fermentum* to the membrane. Adhesive interactions between bacteria and MFG were investigated using SEM and indicated by adhesive linkages between fresh globules and bacteria indicating interaction with potential membrane binding sites [43, 44]. In contrast, fewer bacteria were observed around mastitic globules including fewer adhesive linkages possibly due to altered globule surface due to damage to globule producing machinery in mastitis and/or damage caused by pro-inflammatory cytokines [45] including increased lipolysis and proteolysis resulting in damaged globules surface [18]. Fewer bacteria interacting with mastitic globules could also possibly be due to absence and/or unavailability of adhesive sites due to the blockage by casein molecules [28, 46]. Change in surface charge could repel the bacteria resulting in decreased adhesion [47]. Furthermore, phospholipid sites on globule membrane could play a role in modulating the binding of *L*. *fermentum* to globules. For instance, PAS6/7, glycoprotein known to bind to bacteria, is protected from pepsin digestion by membrane lipids [9, 48].

## Conclusion

The present study reports a comparative study of surface morphological differences between fresh and mastitic MFGs. Lower XO activity in fresh relative to mastitic globules indicate possible alteration in membrane architecture in mastitis resulting in dissociation of membrane-bound enzyme. Effect of globule membrane on adhesion with LAB revealed preferential binding of *L*. *fermentum* to fresh globule membrane, highlighting the significance of undamaged MFGM and the presence of possible adhesive sites compared to mastitic globules. Our study provides further insights into the influence of intact globule membrane morphology on the interaction with probiotic bacteria as potential natural carriers to the intestine and possibly could play a pivotal role in shaping gut microbial population and development of immune system compared to damaged globules.
